# Aptamer Profiling of A549 Cells Infected with Low-Pathogenicity and High-Pathogenicity Influenza Viruses

**DOI:** 10.3390/v11111028

**Published:** 2019-11-05

**Authors:** Kevin M. Coombs, Philippe F. Simon, Nigel J. McLeish, Ali Zahedi-Amiri, Darwyn Kobasa

**Affiliations:** 1 Department of Medical Microbiology & Infectious Diseases, University of Manitoba, Winnipeg, MB R3E 0J9, Canadazahediaa@myumanitoba.ca (A.Z.-A.); darwyn.kobasa@canada.ca (D.K.); 2 Manitoba Centre for Proteomics & Systems Biology, University of Manitoba, Winnipeg, MB R3E 3P4, Canada; 3 Children’s Hospital Research Institute of Manitoba, University of Manitoba, Winnipeg, MB R3E 3P4, Canada; 4 Special Pathogen Program, National Microbiology Laboratory, Public Health Agency of Canada, Winnipeg, MB R3E 3R2, Canada

**Keywords:** emerging viruses, RNA virus infection, proteomics, aptamers, SOMAScan^®^

## Abstract

Influenza A viruses (IAVs) are important animal and human emerging and re-emerging pathogens that are responsible for yearly seasonal epidemics and sporadic pandemics. IAVs cause a wide range of clinical illnesses, from relatively mild infections by seasonal strains, to acute respiratory distress during infections with highly pathogenic avian IAVs (HPAI). For this study, we infected A549 human lung cells with lab prototype A/PR/8/34 (H1N1) (PR8), a seasonal H1N1 (RV733), the 2009 pandemic H1N1 (pdm09), or with two avian strains, an H5N1 HPAI strain or an H7N9 strain that has low pathogenicity in birds but high pathogenicity in humans. We used a newly-developed aptamer-based multiplexed technique (SOMAscan^®^) to examine >1300 human lung cell proteins affected by the different IAV strains, and identified more than 500 significantly dysregulated cellular proteins. Our analyses indicated that the avian strains induced more profound changes in the A549 global proteome compared to all tested low-pathogenicity H1N1 strains. The PR8 strain induced a general activation, primarily by upregulating many immune molecules, the seasonal RV733 and pdm09 strains had minimal effect upon assayed molecules, and the avian strains induced significant downregulation, primarily in antimicrobial response, cardiovascular and post-translational modification systems.

## 1. Introduction

Influenza A virus (IAV) is a member of the family *Orthomyxoviridae.* IAV has been responsible for numerous yearly epidemics and for at least four pandemics during the past ~100 years; estimates are that more than 100 million people have died from infection during this time period [1,2]. IAV is a small enveloped virus with a genome of eight segments of negative-sense single-stranded RNA that encode 10–15 proteins, depending on the virus strain [3,4,5]. IAV is serologically categorized by two surface proteins: The hemagglutinin (HA), of which there are currently 18 types (H1–H18), and neuraminidase (NA), of which there currently are 11 types (N1–11) [3,6].

Virtually all H/N combinations have been identified in water fowl [7,8], the generally-accepted reservoir, except for the recent identification of H17N10 and H18N11 in bats [6], but only a small number of H/N types are known to circulate or have circulated in humans; H1N1 (1918 “Spanish Flu” and the pandemic H1N1 2009 strains), H2N2 and H3N2. There has also been a sporadic but highly lethal spread of H5N1 and H7N9 from birds to humans [9]. Several anti-viral strategies, including small molecule inhibitors and yearly re-formulated vaccines, have been developed to combat IAV, but the virus′ extensive genetic plasticity, caused by an error-prone polymerase and capacity to mix genetic content, often leads to resistance to virus-targeted anti-viral strategies. Because all viruses are obligate parasites, and must therefore make widespread use of host cell machinery, an alternate anti-viral strategy that is being explored would be to elucidate host factors that are required by, and modulated by, the virus, for its successful pathogenicity and propagation.

We [10,11,12,13] and others [14,15,16,17] have used mass-spectrometry (MS)-based non-biased quantitative strategies to measure how IAV affects the host cell proteome. Each of these assays identified thousands of cellular proteins, and collectively, these studies have determined dysregulation of various pathways, such as acetylation, cell structure, defense responses, protein binding, responses to stress, stimulus and virus, alternative splicing, localization, transport, protein binding and nucleoside, nucleotide and nucleic acid metabolism. However, these non-biased global types of strategies have a few limitations. First, since the more abundant proteins are likely to be identified by MS, the less abundant proteins, many of which play key functions, are more likely to be missed. In addition, there usually is less than 100% overlap between replicate MS-identified samples. Multiplex protein arrays have the potential to overcome such limitations, but most are antibody-based and currently limited to a few hundred analytes.

A newly developed proteomics technology, called SOMAscan^®^, and developed by SomaLogics, Inc. (Boulder, CO, USA) provides an alternate strategy to simultaneously sample >1300 specific proteins of interest [18,19,20] in up to ~90 samples. SOMAmers^®^ are chemically modified slow off-rate aptamers (short nucleotides) to stabilize protein binding capacity. We used this technology to complement our earlier quantitative MS-based studies and identified numerous low-abundant proteins, including cytokines and chemokines that are differentially dysregulated by IAV associated with no or mild human infection, compared to H5N1 and H7N9 strains associated with highly pathogenic human infection.

## 2. Materials and Methods

### 2.1. Cells and Viruses

#### 2.1.1. Cells

Human lung A549 cells (ATCC # CCL-185, Manassas, VA, USA) were routinely cultured in Dulbecco′s modified MEM (DMEM) supplemented with 0.2% (*w*/*v*) glucose, non-essential amino acids, sodium pyruvate, 2 mM l-glutamine, and 10% Fetal bovine serum (FBS; Gibco, Grand Island, NY, USA). Madin-Darby canine kidney (MDCK) cells (ATCC # CCL-34) were cultured similarly, but in completed DMEM supplemented with 5% FBS. Cells were grown as monolayers in 5% CO_2_ and passaged by trypsinization 2–3 times each week.

#### 2.1.2. Viruses

Influenza virus strains A/PR/8/34(H1N1; PR8); A/Canada/RV733/2003(H1N1; RV733), an A/New Caledonia/20/1999-like clinical isolate, the 2009 H1N1 pandemic A/Mexico/INDRE4487/2009 (H1N1; pdm09) strain, A/Indonesia/05/2005 (H5N1), and A/Anhui/1/2013 (H7N9) were amplified, concentrated by ultracentrifugation, and titered in MDCK cells by a standard plaque assay or TCID_50_ procedures [13,21]. All manipulations of live H5N1 and H7N9 viruses were performed in a Respiratory BSL-3 facility, using all Public Health Agency of Canada (PHAC) guidelines and SOPs.

#### 2.1.3. Infections

A549 cells were infected, or sham (mock)-infected with diluent, with each of the above viruses at an MOI of 5 when cells were ~90% confluent. Mock and infected cells were harvested at 24 h post-infection (hpi). Aliquots of all infections were saved for plaque titration or TCID_50_ determination to confirm infection. Mock and infected cells were washed, lysed in MPER solubilization buffer (Pierce, Waltham, MA, USA) supplemented with 1 × HALT Protease Inhibitor (Pierce) and protein concentrations determined using the bicinchoninic acid method.

### 2.2. Quantitative SOMAscan^®^ Analyses

Cell lysate protein concentrations were adjusted to 200 ng/μL, and 70 µL of each was analyzed in-house on a SomaLogics^®^-licensed SOMAscan^®^ version 1.3 platform in the Manitoba Centre for Proteomics and Systems Biology as described [22,23]. Briefly, SOMAscan^®^ is a new proteomic tool that uses SOMAmers^®^, DNA Slow Off-rate Modified Aptamers (22). These modified nucleotides are used because they bind to specific human proteins. SOMAmers^®^ bind proteins in their native state, and are measured on DNA microarray chips. SOMAmers^®^ measure fM–μM protein quantities. The SOMAscan^®^ version 1.3 simultaneously measures 1310 distinct proteins in up to 88 samples [22]. Two biologic replicates of PR8 and RV733, and three biologic replicates of Mock-, pdm09-, H5N1- and H7N9-infected samples were collected at 24 hpi (=14 total samples) and were analyzed at the same time in a single SOMAscan^®^ 96-well plate. Results are reported as relative fluorescent units (RFUs) for every protein, and these RFUs are directly proportional to the quantities of each target protein in the original samples, confirmed by standard curve generation for every protein-SOMAmer pair [22]. Differences between Mock and Influenza A virus (IAV)-infected RFU values were analyzed as described below in next section.

### 2.3. Statistical and Bioinformatics Analyses

RFU values for each of the 1310 analyzed proteins in each biologic replicate were imported into Excel. Values were Log_2_-transformed and fold-changes determined for each protein in infected samples compared to mock samples. Fold-change significances were tested both by Students t-test (2 tails) and by Z-score analysis, as described [10,23]. Briefly, all fold-changes found not to be significant by the *t*-test were re-tested by the Z-score. This was done by determining the number of standard deviations each protein′s value was from the population mean. Any protein′s Z-score was deemed significant if the average Z-score was >1.96σ or <−1.96 σ, the Z-score satisfied this criterion in at least 2 replicates and trended >½ the same direction in one or fewer replicates. Thus, Z-scores had to be beyond the ±1.96σ limit in both of the PR8 and RV733 analyses. After compiling all proteins deemed significant, we also analyzed levels of fold-changes of the significant proteins, and as described below, applied a fold-change cut-off of 1.50-fold dysregulation (≥1.50-fold if up-regulated, or ≤0.667-fold if down-regulated) to these proteins for added stringency and to maintain workable numbers of proteins for subsequent bioinformatics and pathway analyses.

## 3. Results

### 3.1. Dysregulation of A549 Proteins Determined Using SOMAmers

A549 cell lysates of IAV- and mock-infected cells were collected 24hpi from two (PR8 and RV733) or three (mock and all other IAV clones) biologic replicates and analyzed with a SomaLogic^®^ version 1.3 platform. Each of 1310 protein analytes was examined and relative quantities determined. Values for each of the infected samples were normalized to the mock samples from the same replicates, and these comparative values were then analyzed. More than 500 proteins were significantly dysregulated by infection (*p*-value < 0.05 and/or Z-score > 1.96σ or <−1.96σ), and far more proteins were dysregulated by both of the H5N1 and H7N9 avian strains than by any of the H1N1 strains (Table 1).

**Figure 1:**
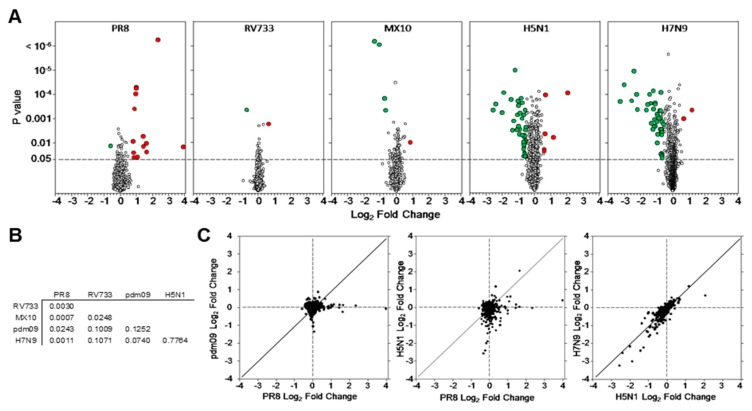
Protein dysregulation characteristics of IAV-infected A549 cells. (**A**) Volcano plots of dysregulated proteins. The dashed horizontal line corresponds to *p*-value 0.05. Proteins significantly upregulated ≥1.5-fold (≥+0.585 Log_2_) are indicated with larger red circles and proteins downregulated ≥1.5-fold (≤−0.585 Log_2_) are indicated with larger green circles. (**B**) Pairwise R^2^ comparisons of indicated virus-infected cellular proteins, with (**C**) Individual proteins of selected pairs graphically presented.

Most of the dysregulated proteins were affected <25%; thus, we considered more stringent parameters and chose fold-change cut-offs of 1.5-fold (=down-regulated to 0.667 of mock) along with significance. Thus, proteins that were dysregulated >1.5-fold, but not considered significant by either *p*-value or Z-score, because of a substantial variability in replicate values, were excluded from the subsequent analysis. Using these parameters, we identified 76 proteins that were significantly dysregulated by infection with any of the tested viruses (Table 1 and Table 2; Figure 1A). Five or fewer proteins were significantly dysregulated by RV733 and the pdm09 strains, 15 were dysregulated by PR8, with all but one being significantly upregulated, and 38 and 47 were dysregulated by H5N1 and H7N9, respectively, with the vast majority of these proteins downregulated.

Several immune-regulating proteins, including ISG15, OAS1 and STAT1 were upregulated by PR8 infection (Table 2), consistent with our previous MS-based proteomic analyses [10]. Thrombopoietin and neural cell adhesion molecule L1 were the only proteins upregulated by RV733 and pdm09, respectively. Few proteins were upregulated by the avian IAV strains in the panel of 1310 SOMAmers. C-C motif chemokine 5 (CCL5) and IL-8 (CXCL8) were both upregulated by both H5N1 and H7N9 and a few other proteins were upregulated by PR8 and H5N1 (Table 2). Pairwise comparisons revealed little correlation between the identities of proteins dysregulated by most virus strains, but an R^2^ value of 0.77 was determined when H5N1-dysregulated proteins were compared to H7N9-dysregulated proteins (Figure 1B,C). Many common proteins were similarly statistically downregulated by both H5N1 and H7N9 and some of these, including Fibronectin and Laminin, had been identified previously as downregulated by iTRAQ-based quantitative MS [13].

### 3.2. H5N1 and H7N9 Induce More Profound Proteomic Dysregulation

**Figure 2:**
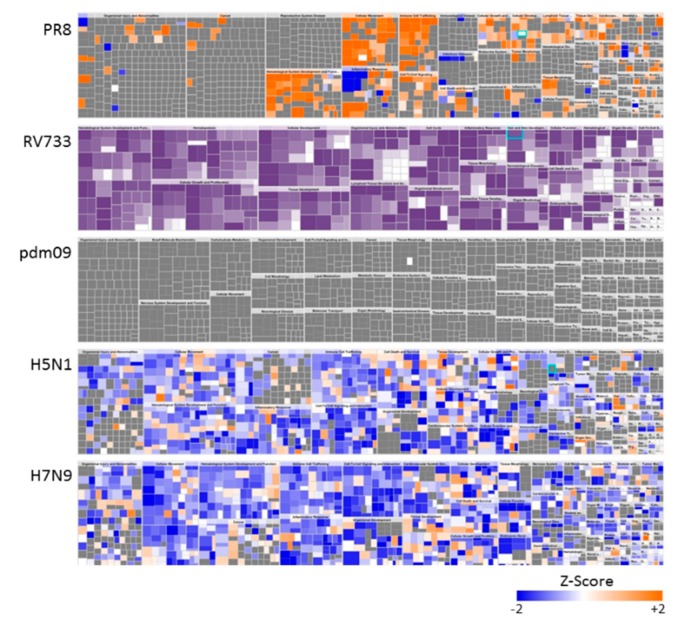
Global dysregulation of A549 proteome by various IAV. All significantly dysregulated A549 proteins altered by >1.33-fold were uploaded to the Ingenuity Pathway Analysis (IPA) tool and each virus’ “Disease Functions” mapped. Orange color represents upregulated proteins within specific disease clusters, blue represents downregulated proteins, grey and purple represent proteins not significantly regulated, and white were not measured by the SOMAmers.

Expression values for all 1310 proteins in each infection were uploaded into the Ingenuity Pathway Analysis (IPA) tool. For this we expanded consideration of dysregulated proteins to those significantly dysregulated >1.33-fold to increase the number of analyzed molecules to nearly 100. As reflected by the numbers of dysregulated proteins induced by each virus (Table 1 and Table 2; Figure 1A), PR8-infected A549 cells demonstrated a moderately global positive Z-score, RV733 and pdm09-infected cells showed no significant positive or negative Z-score, and both H5N1- and H7N9-infected cells showed overall negative Z-scores (Figure 2).

**Figure 3:**
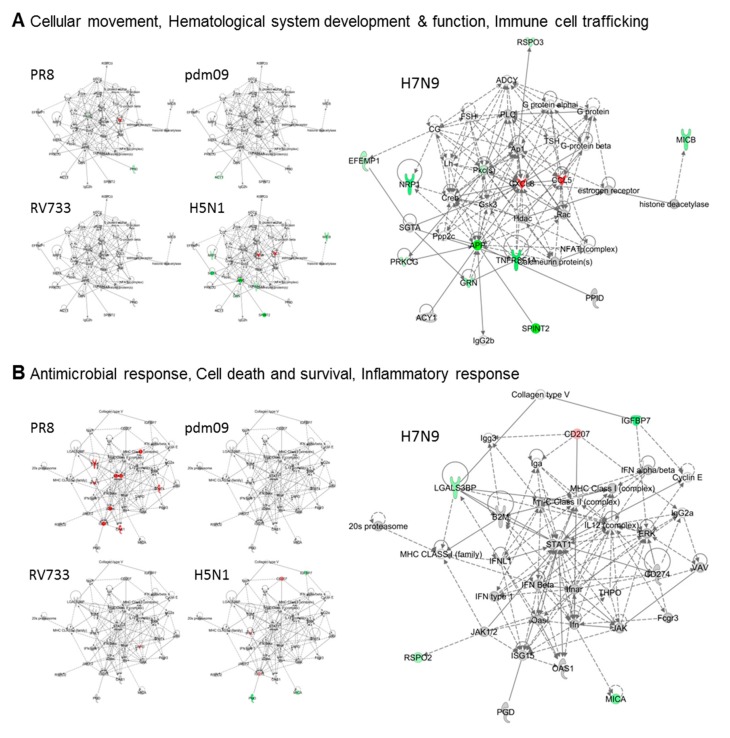

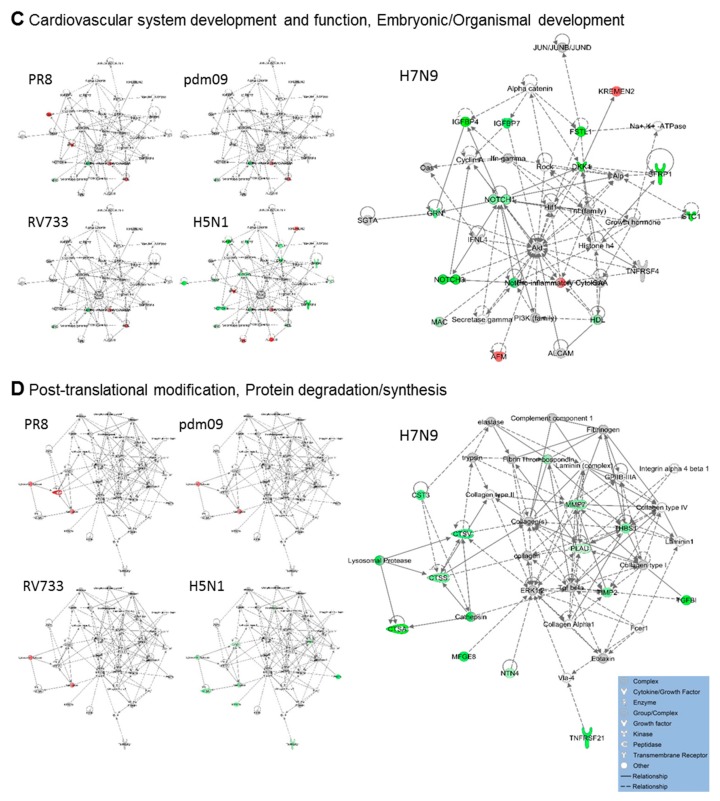
Significant differentially regulated A549 protein networks. (**A**–**D**) correspond to indicated IPA-defined Networks. Proteins and their levels of regulation were imported into the IPA^®^ tool and interacting pathways were constructed under default settings. Four of the top dysregulated A549 cell networks that contain 12 or more “focus” molecules (molecules significantly up- or downregulated) and that have network scores ≥20 are depicted. Red: Significantly upregulated proteins; Pink: moderately upregulated proteins; Gray: proteins within the SOMAscan panel, but whose expression was not significantly up- or downregulated by indicated infection; Light Green: moderately downregulated proteins; Dark Green: significantly downregulated proteins; White: proteins known to be in network, but not covered within SOMAscan panel. Dashed lines represent predicted or indirect interactions; solid lines represent direct known interactions.

These data imply that overall, the seasonal RV733 strain and the pdm09 strain have relatively mild effect, as measured by this small set of SOMAmers, the PR8 strain has an overall activation effect, primarily of immune-modulated and cellular movement molecules, and the H5N1 and H7N9 strains have dramatic inhibitory effects upon multiple cellular processes. IPA network analyses also revealed differences in how each virus affected common cellular networks. The four highest scoring Networks, based on numbers of focus molecules, were the Cellular movement, hematological system development and function, immune cell trafficking, the Antimicrobial response, cell death and survival, inflammatory response, the Cardiovascular system development and function, embryonic development and organismal development and the Post-translational modification, protein degradation and protein synthesis networks. The Cellular movement, hematological system development and function, and the immune cell trafficking network was mildly affected by PR8. No proteins in this network were affected by RV733 or pdm09. Apart from CCL5 and CXCL8, which were both upregulated by both H5N1 and H7N9, many proteins in this network were significantly downregulated by the two avian IAV strains (Figure 3A). The Antimicrobial response, cell death and survival inflammatory response network was most significantly affected by PR8 infection, with numerous upregulated proteins (Figure 3B). This network also was mildly affected by RV733 infection, but not by pdm09 infection. Three focus molecules in this network (CD207, ISG15 and IFNL1) were upregulated and three (IGFBP7, MICA and PGD) were downregulated by H5N1 infection. CD207 was also upregulated and MICA also was downregulated by H7N9 infection, and additionally, LGALS3BP and RSPO2 were downregulated by H7N9 infection. One or more proteins in the Cardiovascular system development and function, embryonic development and organismal development network were upregulated and one or more were downregulated by every virus tested. However, many more proteins (such as FSTL1, IGFBP4, NOTCH and STC) were downregulated by the H5N1 and H7N9 viruses than by any of the H1N1 viruses. Furthermore, HDL was slightly upregulated in every H1N1 network, but downregulated in both the H5N1 and H7N9 networks. Finally, there also were substantial differences in the Post-translational modification, protein degradation and protein synthesis network. Many more proteins (such as CTSA, CTSV, MFGE8 and TNFRSF21) were downregulated by the H5N1 and H7N9 viruses than by any of the H1N1 viruses. Furthermore, cathepsin was slightly upregulated in every H1N1 network, but downregulated in both the H5N1 and H7N9 networks.

**Figure 4:**
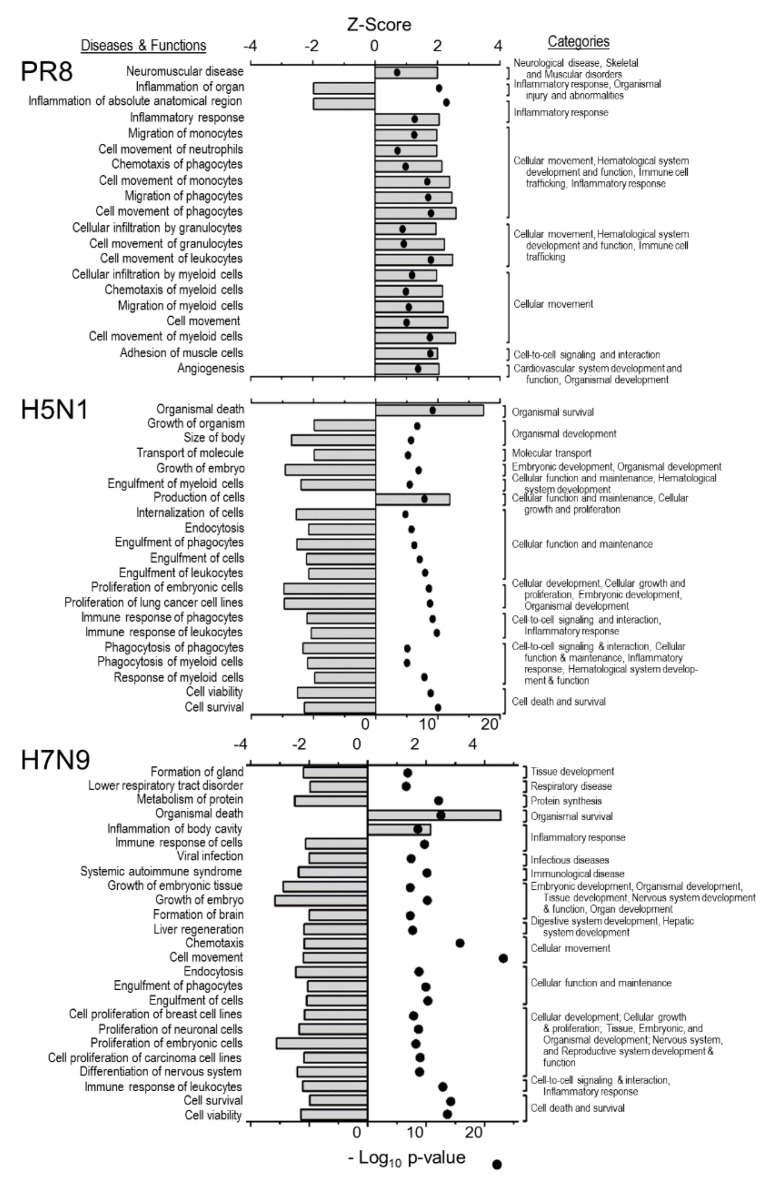
IPA-predicted most affected bio-functions by differentially-expressed proteins by each IAV strain. The default IPA Z-score settings for activation (+1.96σ) and inhibition (−1.96σ) were used. Z-scores are indicated on upper X-axes of each graph and by horizontal bars, and −Log_10_ *p*-values (bottom X-axes) are depicted by ●. There were no significantly dysregulated bio-functions by RV733 or pdm09.

Various bio-functions also were examined using the IPA^®^ default settings for these analyses (Figure 4). Bio-function activation is assumed for Z-scores > 1.96σ, and bio-function inhibition is assumed for Z-scores < −1.96σ. All of these indicated bio-functions also had significant *p*-values. PR8 activated many bio-functions, including cellular movement categories, inflammatory response, and angiogenesis, and inhibited few bio-functions; organ inflammation and anatomical organ inflammation.

The avian IAV inhibited far more bio-functions, including cell survival and viability; endocytosis and engulfment, immune response and the proliferation of numerous cell types. The avian IAV strains activated few bio-functions, one of which was organismal death.

**Figure 5:**
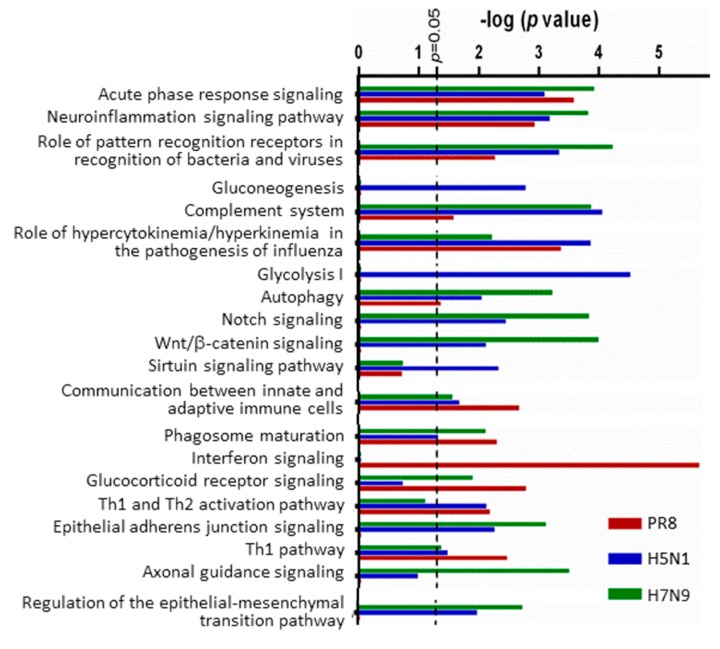
Comparison of cellular canonical signaling pathways affected by various IAV strains. Only the top 20 IPA^®^-identified canonical pathways and their indicated *p*-values are shown. RV733 and pdm09 did not activate any of these pathways.

We then used IPA to compare various cellular canonical signaling pathways (Figure 5). The virus strains that induced the most profound cellular responses (PR8, H5N1 and H7N9) affected many canonical pathways. PR8 uniquely significantly activated interferon signaling, which has been reported previously [10,11], while H5N1 uniquely inhibited gluconeogenesis and glycolysis I, and all three strains had significant effects upon multiple pathways, including acute phase response signaling, neuroinflammation, role of pattern recognition of viruses, the complement system, and autophagy. The avian strains had the most dramatic effects upon multiple canonical pathways, including Notch signaling, Wnt/β-catenin signaling, epithelial adherens junction signaling and regulation of epithelial-mesenchymal transition. Thus, collectively and overall, the H5N1 and H7N9 avian IAV strains induced more profound inhibitory cellular responses than any of the H1N1 strains, consistent with our [13] and other′s [17] studies.

## 4. Discussion

Influenza A virus (IAV) remains a significant human pathogen that is constantly emerging and re-emerging. The virus’ genetic make-up of eight segments of single-stranded RNA allows for significant genetic plasticity. Like other RNA viruses that lack genetic proof reading, the highly error-prone RNA-dependent RNA polymerase leads to a high mutation rate (=antigenic drift), and the segmented genomes allows for segment mixing during co-infections, leading to emergence of new isolates (=antigenic shift). Thus, vaccines need to be reformulated each year, and the process of attempting to anticipate future isolates often leads to vaccine mismatch [24], which may be exacerbated by an individual′s immune history [25]. A limited repertoire of anti-viral compounds that either inhibit viral uncoating or viral release, have been approved, but viral mutation quickly arises during outbreaks [26,27]. Thus, there has been increasing interest to identify host factors that the virus may require for replication and pathogenicity to complement strategies that only target the virus. A number of studies, including genome-wide RNAi screens, mRNA microarray screens and yeast 2-hybid assays have identified >1500 cellular targets worthy of further analysis [28,29,30,31].

Numerous recent studies have used quantitative MS-based assays to probe the cellular proteome after perturbation by virus infection. These include 2-dimensional differences in gel electrophoresis (2D-DIGE) (ex. [32,33]), stable isotopic labeling by amino acids in cell culture (SILAC) (ex. [10,34,35]), isobaric tags for relative and absolute quantitation (iTRAQ) or tandem mass tags (TMT) (ex. [13,36,37,38]), and label-free methods [39,40,41]. Each of these non-biased techniques provides information about thousands of proteins within complex mixtures, including cell extracts (reviewed in [42,43,44]). However, each of these provide only a small sampling of the entire cellular proteome. Most of these methods detect and measure the most abundant proteins within the mixture.

Furthermore, there is less than 100% overlap between any two sample runs, whether biologic runs or technical replicates of the same sample. Alternate multiplex assays that target specific proteins have been developed and include the MyriadRBM^®^ and Luminex^®^ platforms. Many of these are limited to fewer than 100 proteins that can be simultaneously assayed and measured, although a few, such as those offered by RayBiotech^®^, are reported to detect and measure several hundred proteins. Thus, at the time we initiated these studies, we selected an aptamer-based assay reported to reliably detect and measure more than 1000 proteins. These slow-off-rate modified aptamers (SOMAmers^®^) were developed by SomaLogics, Inc., (Boulder, CO, USA) [18,19,20], and have been used to examine cancer biomarkers [45], Alzheimer′s disease biomarkers [46] and biomarkers in IAV-infected clinical nasal secretions [47]. Our pilot studies with the first available version, capable of detecting 1128 proteins, reliably measured differences in influenza PR8-infected A549 cells over a time course. We subsequently used the next-generation SOMAscan version 1.3, which measures >1300 proteins, to measure Zika virus-induced proteomic alterations in Vero cells [23] and in U251 astrocytoma cells [48]. Application of this targeted aptamer-based approach, which was designed primarily to detect low-abundance proteins, thus provides a beneficial complementary approach to the non-biased MS-based approaches that tend to measure more abundant proteins. Comparative analyses of dysregulated proteins we identified in this study to proteins identified in one of our earlier MS-based studies [10,34,35] showed generally good agreement. Two proteins (ISG15 and β-2-microglobulin) were identified as upregulated by both methods, 75 proteins were determined to not be significantly regulated by both techniques and no proteins were identified as significantly upregulated by one method, but significantly downregulated by the other. Thus, this newer aptamer-based multiplexed system, designed primarily to detect and measure lower abundant proteins, provides complementary data to the more commonly-used MS-based approaches and to assay numerous proteins not normally detected by quantitative MS.

The current study assayed 2–3 biologic replicates of five different IAV strains, each compared to mock-infected samples (a total of 14 samples). These virus strains represent a lab-adapted H1N1 strain (PR8), a mild seasonal H1N1 strain related to the A/New Caledonia/20/1999-like clinical isolate (RV733), the 2009 H1N1 pandemic strain (pdm09), and two strains that show significantly higher pathology in human patients; the H5N1 “Bird flu” and the H7N9 strain. All tested virus strains affected the A549 cellular proteome, upregulating some proteins and downregulating other proteins. Although none of the 1310 measurable common cellular proteins was significantly dysregulated by all five viruses, some proteins were similarly dysregulated by multiple viruses. For example, CCL5 was upregulated by PR8, H5N1 and H7N9, and PGAM1 was significantly downregulated by pdm09, H5N1 and H7N9. The five viruses generated three overall patterns of dysregulation, at least as measured by the 1310 proteins that can be assayed by the SOMAscan. PR8 caused an overall general activation, primarily of antimicrobial inflammatory and immune response, as reflected by a large upregulation of more than a dozen proteins, including ISG15, STAT1, OAS1 and downregulation only of PPID. PR8 is a highly lab culture-adapted strain passaged multiple times in mice that is highly virulent in mice, but extremely attenuated in humans. RV733 and pdm09 had very little effect in our A549 cells, as we found in a previous quantitative MS study [13], and are relatively low virulence, despite being well adapted to growth in humans. Both the H5N1 and H7N9 viruses, which demonstrate the third pattern (significant downregulation of many more proteins than the other strains, particularly in the antimicrobial response, cardiovascular, and post-translational modification networks), and significant downregulation of many common proteins, are poorly adapted to humans because of their receptor specificity, but when successfully delivered into the lower human respiratory tract, they can be highly virulent. This more profound proteomic effect by these avian-derived viruses also was observed in quantitative transcriptomic [49] and MS studies [13].

Genome-wide RNAi screens and mRNA microarray screens identified >1500 cellular genes and proteins influenza virus may depend upon [28,29,30,31]. For example, Konig and colleagues identified 295 genes that were required for influenza virus replication as defined by replicase activity [29] and Karlas and colleagues identified 287 genes required for replication of two different influenza viruses, including the pdm09 strain [28]. Collectively, these two RNAi screens, also carried out in A549 cells, identified 552 potential genes important for influenza replication, with 30 genes found in common. The low level of overlap between these various RNAi screens has been previously noted [50]. We assessed potential overlap between genes identified in the two Karlas and Konig A549 studies with proteins we could detect and measure in A549 cells with our SOMAscan^®^ panel. Only four genes/proteins (JUN, KPNB1, MAP2K3 and MDM2) overlapped in all three datasets, and of these, only KPNB1 was significantly altered according to SOMAscan^®^; downregulated 1.48-fold by H5N1 infection. Thirty-one additional genes identified by Karlas are in the SOMAscan^®^ panel; of these, 14 were significantly dysregulated by at least one of our tested viruses. Seven proteins were significantly dysregulated by both H5N1 and H7N9, but <1.35-fold. Only one protein, B2M, was dysregulated >1.35-fold; it was upregulated 2.7-fold and only by PR8 infection. Forty genes identified by Konig, including the four found in all three datasets, are in the SOMAscan^®^ panel; of these, 12 were significantly dysregulated by at least one of our tested viruses. FGFR4 was dysregulated, but less than 20%, by three viruses; pdm09, H5N1 and H7N9. Only two of the 14 proteins were dysregulated >1.5-fold; APP was downregulated 3.7-fold by H5N1 and 8-fold by H7N9, and FGFR1 was downregulated 2.1-fold by H7N9 infection. The 71 genes/proteins identified by Karlas and/or Konig and that were present in our SOMAscan^®^ panel represent proteins involved in numerous diverse functions, including cytokines, enzymes, growth factors, transmembrane receptors and many kinases, as do many of the proteins newly identified in this SOMAscan. Thus, these different methods identify a large number of cellular genes and proteins that should be more extensively analyzed as potential targets to ameliorate influenza virus infection.

Some of our observed dysregulated proteins also were observed in a clinical analysis using the SOMAscan platform. Marion and colleagues found some of their most significantly differentially expressed proteins were CTSD, KLK7, MFGE8, MAPK9 and CD27 [47]. MFGE8 (Lactadherin) was significantly downregulated only by H5N1 and H7N9 in our study. Although CTSD (cathepsin D), KLK7 (Kallikrein-7), MAPK9 (Mitogen activated protein kinase 9) and CD27 antigen were not significantly affected by any of our tested viruses, other cathepsin isoforms, CTSV (cathepsin L2) was significantly downregulated by H7N9, and CTSS was significantly upregulated by PR8, KLK5 was upregulated by pdm09, but only 1.4-fold, These differences probably relate to study design; Marion assessed clinical nasal swabs and we examined A549 cell extracts.

The A549 cell is a transformed adenocarcinoma cell line derived from an explanted tumor from a 58-year-old Caucasian male. While this study provides some important information about how multiple IAV strains induce changes in the cellular proteome of these cells, it will be important to perform similar assays in more physiologically-relevant primary cells to allow the comparison and possible identification of common cellular processes that might be amenable to therapeutic intervention.

## 5. Conclusions

In conclusion, we used a targeted aptamer-based approach to complement earlier quantitative MS approaches to compare host cell responses to viruses that have differential host pathology. We found that the culture-adapted PR8 strain had an overall activation of immune molecules, the mild seasonal and pdm09 human strains had little effect upon the molecules targeted by the SOMA panel, and the avian H5N1 and H7N9 strains, that are much more pathogenic in humans, had the most dramatic proteomic responses, these upregulating a few tested molecules, but inhibiting many more key cellular processes.

## Figures and Tables

**Table 1:** Numbers of significantly dysregulated A549 proteins induced by each Influenza A virus (IAV) strain.

Number That Are Significant	Total Unique	PR8	RV733	pdm09	H5N1	H7N9
and fold-change > 1.000	510	33	20	67	194	133
and fold-change < 0.9999		15	7	38	166	168
and fold-change > 1.250	128	17	1	7	15	10
and fold-change < 0.8000		1	1	14	57	65
and fold-change > 1.333	98	15	1	6	8	6
and fold-change < 0.7500		1	1	6	45	56
and fold-change > 1.500	76	14	1	1	6	2
and fold-change < 0.6667		1	1	4	32	45
and fold-change > 2.000	33	8	0	0	2	1
and fold-change < 0.5000		0	0	2	11	19
and fold-change > 3.000	11	4	0	0	1	0
and fold-change < 0.3333		0	0	0	4	7
and fold-change > 5.000	6	2	0	0	0	0
and fold-change < 0.2000		0	0	0	2	4

Significance determined by *T*-test and Z-score as described in Materials & Methods. The 76 specific proteins significantly dysregulated >1.5-fold are listed in Table 2.

**Table 2:** A549 proteins dysregulated by infection by indicated influenza strains.

EntrezGene Symbol	Protein	Fold-Change Compared to Sham-Infected				
		H1N1 Viruses				
		PR8	RV733	pdm09	H5N1	H7N9
***Up-Regulated Proteins***						
ISG15	Ubiquitin-like protein ISG15	**15.9**	1.03	0.96	1.33	1.10
OAS1	2′-5′-oligoadenylate synthase 1	**5.06**	0.99	1.08	0.97	0.96
CCL5	C-C motif chemokine 5	**3.11**	1.06	1.09	**4.18**	**1.62**
STAT1	Signal transducer and activator of transcription 1-alpha/beta	**3.09**	1.26	0.85	1.02	1.00
B2M	Beta-2-microglobulin	**2.72**	0.97	1.11	1.12	0.99
APOL1	Apolipoprotein L1	**2.68**	0.98	0.99	0.99	0.92
CD274	Programmed cell death 1 ligand 1	**2.09**	1.06	1.05	1.27	1.13
CTSS	Cathepsin S	**2.00**	0.99	1.05	0.79	**0.59**
SERPINE1	Plasminogen activator inhibitor 1	**1.99**	1.00	1.10	0.89	0.70
IFNL1	Interferon lambda-1	**1.97**	1.00	0.99	**1.52**	1.21
F2	Thrombin	**1.84**	1.03	1.00	0.97	0.89
PLAUR	Urokinase plasminogen activator surface receptor	**1.80**	0.98	1.05	1.05	0.85
MDK	Midkine	**1.80**	1.01	0.97	1.02	0.93
CFB	Complement factor B	**1.75**	1.04	1.03	1.03	0.96
THPO	Thrombopoietin	1.01	**1.58**	1.01	1.00	1.10
L1CAM	Neural cell adhesion molecule L1	1.21	1.28	**1.83**	**1.57**	1.47
CXCL8	Interleukin-8	1.26	1.00	0.99	**2.26**	**2.30**
CD207	C-type lectin domain family 4 member K	1.10	1.05	1.06	**1.61**	1.38
F9	Coagulation factor IX	2.21	1.03	1.14	**1.53**	1.29
***Down-regulated proteins***						
PPID	Peptidyl-prolyl cis-trans isomerase D	**0.66**	1.15	0.95	0.97	1.03
TGM3	Protein-glutamine gamma-glutamyltransferase E	0.88	**0.61**	1.21	0.75	0.90
PGAM1	Phosphoglycerate mutase 1	1.05	0.83	**0.39**	**0.54**	**0.50**
MDH1	Malate dehydrogenase, cytoplasmic	1.01	1.02	**0.48**	0.59	0.83
LDHB	L-lactate dehydrogenase B chain	0.97	1.19	**0.61**	0.84	1.02
ENO1	Alpha-enolase	0.91	0.96	**0.63**	0.77	0.82
PCSK9	Proprotein convertase subtilisin/kexin type 9	0.80	0.92	1.07	**0.17**	**0.11**
DKK1	Dickkopf-related protein 1	0.85	0.87	0.92	**0.19**	**0.17**
DKK4	Dickkopf-related protein 4	0.88	0.91	0.97	**0.25**	**0.22**
APP	Amyloid beta A4 protein	0.93	0.94	0.99	**0.27**	**0.12**
SPINT2	Kunitz-type protease inhibitor 2	0.93	0.87	0.95	**0.37**	**0.19**
TNFRSF4	Tumor necrosis factor receptor superfamily member 4	1.01	0.53	0.56	**0.39**	0.54
IGFBP4	Insulin-like growth factor-binding protein 4	1.28	0.95	1.06	**0.41**	**0.21**
PGD	6-phosphogluconate dehydrogenase, decarboxylating	0.98	1.12	0.80	**0.44**	0.66
FN1	Fibronectin	1.12	0.94	0.95	**0.47**	**0.40**
TGFBI	Transforming growth factor-beta-induced protein ig-h3	0.87	0.95	0.95	**0.48**	**0.39**
SGTA	Small glutamine-rich tetratricopeptide repeat-containing protein alpha	0.93	1.01	0.65	**0.49**	0.80
GAPDH	Glyceraldehyde-3-phosphate dehydrogenase	0.97	1.00	0.70	**0.50**	0.83
FSTL3	Follistatin-related protein 3	1.49	0.92	1.04	**0.50**	**0.33**
FN1	Fibronectin Fragment 3	1.12	0.95	0.94	**0.53**	**0.47**
CTSA	Lysosomal protective protein	0.75	0.94	1.04	**0.53**	**0.35**
MICB	MHC class I polypeptide-related sequence B	0.88	0.93	1.00	**0.57**	**0.60**
NOTCH3	Neurogenic locus notch homolog protein 3	1.06	0.90	0.95	**0.59**	**0.47**
PKM2	Pyruvate kinase PKM	0.80	1.10	1.38	**0.59**	0.81
LRIG3	Leucine-rich repeats and immunoglobulin-like domains protein 3	1.50	0.87	0.92	**0.59**	**0.63**
MFGE8	Lactadherin	0.91	0.94	1.05	**0.63**	**0.43**
PEX5	Peroxisomal targeting signal 1 receptor	0.98	0.71	0.73	**0.63**	0.75
WNK3	Serine/threonine-protein kinase WNK3	1.10	0.93	0.86	**0.63**	0.77
TNFRSF21	Tumor necrosis factor receptor superfamily member 21	0.96	0.92	0.96	**0.63**	**0.43**
SFRP1	Secreted frizzled-related protein 1	1.27	1.00	0.98	**0.64**	**0.45**
TNFRSF1A	Tumor necrosis factor receptor superfamily member 1A	0.74	0.97	1.09	**0.64**	**0.46**
FSTL1	Follistatin-related protein 1	1.28	0.92	1.02	**0.65**	**0.44**
IGFBP7	Insulin-like growth factor-binding protein 7	1.06	0.96	1.00	**0.65**	**0.53**
NRP1	Neuropilin-1	1.09	0.94	0.89	**0.65**	**0.53**
CSF3R	Granulocyte colony-stimulating factor receptor	1.03	0.75	0.83	**0.66**	0.75
C3	C3a anaphylatoxin des Arginine	1.12	0.96	1.00	**0.66**	**0.61**
CFH	Complement factor H	1.54	1.00	0.96	**0.66**	**0.52**
STC1	Stanniocalcin-1	1.19	0.89	1.05	0.69	**0.37**
FGFR1	Fibroblast growth factor receptor 1	1.02	0.94	1.02	0.69	**0.47**
CTSV	Cathepsin L2	0.83	0.98	1.08	0.74	**0.52**
CST3	Cystatin-C	1.39	0.94	1.10	0.86	**0.55**
PLXNB2	Plexin-B2	1.05	1.02	1.14	0.71	**0.58**
LGALS8	Galectin-8	1.18	0.92	0.92	0.78	**0.59**
NRG1	Neuregulin-1	1.21	1.03	1.24	0.94	**0.60**
GNS	N-acetylglucosamine-6-sulfatase	0.95	1.04	1.09	0.75	**0.60**
MICA	MHC class I polypeptide-related sequence A	0.85	1.04	1.18	0.68	**0.61**
LAMA1 LAMB1 LAMC1	Laminin	0.76	1.09	1.14	0.79	**0.62**
THBS1	Thrombospondin-1	1.05	1.01	1.04	0.68	**0.62**
TIMP2	Metalloproteinase inhibitor 2	0.97	0.93	1.03	0.85	**0.62**
MMP7	Matrilysin	0.93	0.99	1.03	0.93	**0.63**
LCN2	Neutrophil gelatinase-associated lipocalin	0.78	0.96	1.03	0.87	**0.63**
GRN	Granulins	1.04	0.96	1.04	0.67	**0.63**
TFPI	Tissue factor pathway inhibitor	0.80	1.03	1.03	1.10	**0.63**
GFRA1	GDNF family receptor alpha-1	0.77	0.97	0.99	0.75	**0.63**
MET	Hepatocyte growth factor receptor	1.23	1.03	1.11	0.89	**0.64**
KIR2DL4	Killer cell immunoglobulin-like receptor 2DL4	0.92	1.01	0.92	0.77	**0.65**
LGALS3BP	Galectin-3-binding protein	1.49	0.97	1.01	0.83	**0.65**

Values represent protein fold-changes compared to mock-infected. Fold-changes with significance <0.05 and with proteins significantly upregulated ≥1.50-fold are indicated in red, and proteins significantly downregulated ≤0.666-fold are indicated in green. Proteins sorted first by upregulation and from left-most virus column to right-most; then sorted by downregulation from left to right.
